# Health Professional Training and Capacity Strengthening Through International Academic Partnerships: The First Five Years of the Human Resources for Health Program in Rwanda

**DOI:** 10.15171/ijhpm.2018.61

**Published:** 2018-08-06

**Authors:** Corrado Cancedda, Phil Cotton, Joseph Shema, Stephen Rulisa, Robert Riviello, Lisa V. Adams, Paul E. Farmer, Jeanne N. Kagwiza, Patrick Kyamanywa, Donatilla Mukamana, Chrispinus Mumena, David K. Tumusiime, Lydie Mukashyaka, Esperance Ndenga, Theogene Twagirumugabe, Kaitesi B. Mukara, Vincent Dusabejambo, Timothy D. Walker, Emmy Nkusi, Lisa Bazzett-Matabele, Alex Butera, Belson Rugwizangoga, Jean Claude Kabayiza, Simon Kanyandekwe, Louise Kalisa, Faustin Ntirenganya, Jeffrey Dixson, Tanya Rogo, Natalie McCall, Mark Corden, Rex Wong, Madeleine Mukeshimana, Agnes Gatarayiha, Egide Kayonga Ntagungira, Attila Yaman, Juliet Musabeyezu, Anne Sliney, Tej Nuthulaganti, Meredith Kernan, Peter Okwi, Joseph Rhatigan, Jane Barrow, Kim Wilson, Adam C. Levine, Rebecca Reece, Michael Koster, Rachel T. Moresky, Jennifer E. O’Flaherty, Paul E. Palumbo, Rashna Ginwalla, Cynthia A. Binanay, Nathan Thielman, Michael Relf, Rodney Wright, Mary Hill, Deborah Chyun, Robin T. Klar, Linda L. McCreary, Tonda L. Hughes, Marik Moen, Valli Meeks, Beth Barrows, Marcel E. Durieux, Craig D. McClain, Amy Bunts, Forrest J. Calland, Bethany Hedt-Gauthier, Danny Milner, Giuseppe Raviola, Stacy E. Smith, Meenu Tuteja, Urania Magriples, Asghar Rastegar, Linda Arnold, Ira Magaziner, Agnes Binagwaho

**Affiliations:** ^1^Center for Global Health, Perelman School of Medicine, University of Pennsylvania, Philadelphia, PA, USA.; ^2^Office of the Vice-Chancellor, University of Rwanda, Kigali, Rwanda.; ^3^Rwanda Human Resources for Health Program Team, Ministry of Health, Kigali, Rwanda.; ^4^Office of the Dean, School of Medicine and Pharmacy, College of Medicine and Health Sciences, University of Rwanda, Kigali, Rwanda.; ^5^Center for Surgery and Public Health, Brigham and Women’s Hospital, Boston, MA, USA.; ^6^Department of Global Health and Social Medicine, Harvard Medical School, Boston, MA, USA.; ^7^Center for Health Equity, Geisel School of Medicine, Dartmouth College, Hanover, NH, USA.; ^8^Department of Medicine, Geisel School of Medicine, Dartmouth College, Hanover, NH, USA.; ^9^Division of Global Health Equity, Department of Medicine, Brigham and Women’s Hospital, Boston, MA, USA.; ^10^Office of the Principal, College of Medicine and Health Sciences, University of Rwanda, Kigali, Rwanda.; ^11^Department of Surgery, Faculty of Clinical Medicine and Dentistry, Kampala International University - Western Campus, Ishaka, Uganda.; ^12^School of Nursing and Midwifery, College of Medicine and Health Sciences, University of Rwanda, Kigali, Rwanda.; ^13^Office of the Dean and Department of Oral and Maxillofacial Surgery, Oral Pathology and Oral Medicine, School of Dentistry, College of Medicine and Health Sciences, University of Rwanda, Kigali, Rwanda.; ^14^School of Health Sciences, College of Medicine and Health Sciences, University of Rwanda, Kigali, Rwanda.; ^15^Department of Anesthesiology, School of Medicine and Pharmacy, College of Medicine and Health Sciences, University of Rwanda, Kigali, Rwanda.; ^16^Department of Ear, Nose, and Throat, School of Medicine and Pharmacy, College of Medicine and Health Sciences, University of Rwanda, Kigali, Rwanda.; ^17^Department of Internal Medicine, School of Medicine and Pharmacy, College of Medicine and Health Sciences, University of Rwanda, Kigali, Rwanda.; ^18^School of Medicine and Public Health, Faculty of Health and Medicine, University of Newcastle, Newcastle, NSW, Australia.; ^19^Department of General Medicine, Calvary Mater Hospital, Newcastle, NSW, Australia.; ^20^Department of Neurosurgery, School of Medicine and Pharmacy, College of Medicine and Health Sciences, University of Rwanda, Kigali, Rwanda.; ^21^Department of Obstetrics, Gynecology, and Reproductive Sciences, Yale School of Medicine, New Haven, CT, USA.; ^22^Department of Orthopedic Surgery, Rwanda Military Hospital, Kigali, Rwanda.; ^23^Department of Pathology, School of Medicine and Pharmacy, College of Medicine and Health Sciences, University of Rwanda, Kigali, Rwanda.; ^24^Department of Pediatrics, School of Medicine and Pharmacy, College of Medicine and Health Sciences, University of Rwanda, Kigali, Rwanda.; ^25^Department of Mental Health, School of Medicine and Pharmacy, College of Medicine and Health Sciences, University of Rwanda, Kigali, Rwanda.; ^26^Department of Radiology, School of Medicine and Pharmacy, College of Medicine and Health Sciences, University of Rwanda, Kigali, Rwanda.; ^27^Department of Surgery, School of Medicine and Pharmacy, College of Medicine and Health Sciences, University of Rwanda, Kigali, Rwanda.; ^28^Yale School of Medicine, New Haven, CT, USA.; ^29^Department of Pediatrics, Icahn School of Medicine at Mount Sinai, New York City, NY, USA.; ^30^Department of Pediatrics, BronxCare Health System, Bronx, NY, USA.; ^31^Department of Pediatrics, Yale School of Medicine, New Haven, CT, USA.; ^32^Division of Hospital Medicine, Department of Pediatrics, Children’s Hospital Los Angeles, Los Angeles, CA, USA.; ^33^Department of Pediatrics, Keck School of Medicine, University of Southern California, Los Angeles, CA, USA.; ^34^Global Health Leadership Institute, Yale School of Public Health, New Haven, CT, USA.; ^35^Department of Preventive and Community Dentistry, School of Dentistry, College of Medicine and Health Sciences, University of Rwanda, Kigali, Rwanda.; ^36^University of Global Health Equity, Kigali, Rwanda.; ^37^Clinton Health Access Initiative, Boston, MA, USA.; ^38^Clinton Health Access Initiative, Kigali, Rwanda.; ^39^Office of Global and Community Health, Harvard School of Dental Medicine, Boston, MA, USA.; ^40^Department of Oral Health Policy and Epidemiology, Harvard School of Dental Medicine, Boston, MA, USA.; ^41^Department of General Pediatrics, Boston Children’s Hospital, Boston, MA, USA.; ^42^Department of Emergency Medicine, Warren Alpert Medical School of Brown University, Providence, RI, USA.; ^43^Department of Medicine, Warren Alpert Medical School of Brown University, Providence, RI, USA.; ^44^Department of Pediatrics, Warren Alpert Medical School of Brown University, Providence, RI, USA.; ^45^sidHARTe Program, Heilbrunn Department of Population and Family Health, Mailman School of Public Health, Columbia University, New York City, NY, USA.; ^46^Department of Emergency Medicine, Columbia University College of Physicians and Surgeons, New York City, NY, USA.; ^47^Department of Anesthesiology, Geisel School of Medicine, Dartmouth College, Hanover, NH, USA.; ^48^Dartmouth-Hitchcock Medical Center, Lebanon, NH, USA.; ^49^Department of Pediatrics, Geisel School of Medicine, Dartmouth College, Hanover, NH, USA.; ^50^Department of Surgery, Geisel School of Medicine, Dartmouth College, Hanover, NH, USA.; ^51^Duke Hubert-Yeargan Center for Global Health, Durham, NC, USA.; ^52^Department of Medicine, Duke University School of Medicine, Durham, NC, USA.; ^53^Duke Global Health Institute, Durham, NC, USA.; ^54^Duke University Medical Center, Durham, NC, USA.; ^55^Duke University School of Nursing, Durham, NC, USA.; ^56^Department of Obstetrics & Gynecology and Women’s Health, Albert Einstein College of Medicine, New York City, NY, USA.; ^57^Obstetrics & Gynecology and Women’s Health, Montefiore Medical Center, New York City, NY, USA.; ^58^Division of Nursing, Howard University College of Nursing and Allied Health Sciences, Washington, DC, USA.; ^59^University of Connecticut School of Nursing, Storrs, CT, USA.; ^60^New York University Rory Meyers College of Nursing, New York City, NY, USA.; ^61^University of Illinois at Chicago College of Nursing, Chicago, IL, USA.; ^62^Columbia University School of Nursing, New York City, NY, USA.; ^63^Department of Family & Community Health, University of Maryland School of Nursing, Baltimore, MD, USA.; ^64^Global Education and Mentorship, Office of Global Health, University of Maryland School of Nursing, Baltimore, MD, USA.; ^65^Department of Oncology & Diagnostic Sciences, University of Maryland School of Dentistry, Baltimore, MD, USA.; ^66^Office of Global Health, University of Maryland School of Nursing, Baltimore, MD, USA.; ^67^Partnerships, Professional Education, and Practice, University of Maryland School of Nursing, Baltimore, MD, USA.; ^68^Department of Anesthesiology, University of Virginia School of Medicine, Charlottesville, VA, USA.; ^69^Department of Anesthesiology Perioperative and Pain Medicine, Boston Children’s Hospital, Boston, MA, USA.; ^70^Department of Surgery, University of Virginia School of Medicine, Charlottesville, VA, USA.; ^71^Center for Global Health, American Society for Clinical Pathology, Chicago, IL, USA.; ^72^Department of Immunology and Infectious Diseases, Harvard T. H. Chan School of Public Health, Boston, MA, USA.; ^73^Department of Psychiatry, Boston Children’s Hospital, Boston, MA, USA.; ^74^Department of Radiology, Brigham and Women’s Hospital, Boston, MA, USA.; ^75^Global Health and Research Programs, Biomedical Research Institute, Brigham and Women’s Hospital, Boston MA, USA.; ^76^Department of Internal Medicine, Yale School of Medicine, New Haven, CT, USA.; ^77^Institute for Health Policy and Clinical Practice, Dartmouth College, Hanover, NH, USA.; ^78^Office of the Vice-Chancellor, University of Global Health Equity, Kigali, Rwanda.

**Keywords:** Health Professional Training, Human Resource for Health, Institutional Capacity, Strengthening, Academic Partnerships, Rwanda

## Abstract

**Background:** The Rwanda Human Resources for Health Program (HRH Program) is a 7-year (2012-2019) health professional training initiative led by the Government of Rwanda with the goals of training a large, diverse, and competent health workforce and strengthening the capacity of academic institutions in Rwanda.

**Methods:** The data for this organizational case study was collected through official reports from the Rwanda Ministry of Health (MoH) and 22 participating US academic institutions, databases from the MoH and the College of Medicine and Health Sciences (CMHS) in Rwanda, and surveys completed by the co-authors.

**Results:** In the first 5 years of the HRH Program, a consortium of US academic institutions has deployed an average of 99 visiting faculty per year to support 22 training programs, which are on track to graduate almost 4600 students by 2019. The HRH Program has also built capacity within the CMHS by promoting the recruitment of Rwandan faculty and the establishment of additional partnerships and collaborations with the US academic institutions.

**Conclusion:** The milestones achieved by the HRH Program have been substantial although some challenges persist. These challenges include adequately supporting the visiting faculty; pairing them with Rwandan faculty (twinning); ensuring strong communication and coordination among stakeholders; addressing mismatches in priorities between donors and implementers; the execution of a sustainability strategy; and the decision by one of the donors not to renew funding beyond March 2017. Over the next 2 academic years, it is critical for the sustainability of the 22 training programs supported by the HRH Program that the health-related Schools at the CMHS significantly scale up recruitment of new Rwandan faculty. The HRH Program can serve as a model for other training initiatives implemented in countries affected by a severe shortage of health professionals.

## Background

### Rwanda’s Health Workforce Shortage

Rwanda is a small and densely populated country in sub-Saharan Africa about the size of the state of Maryland in the United States and with a population of approximately 11 million. In the aftermath of 1994’s genocide, Rwanda was faced with the daunting challenge of rebuilding its devastated institutions and governance infrastructure, including the health system. Over the past 24 years, Rwanda has aggressively pursued excellence across all sectors (health, education, housing, and finance) by incorporating a strong equity agenda into the national development plan, which was published in 2000 and is known as “Vision 2020.” This plan has paved the way for Rwanda’s subsequent development achievements, especially in health, which have included some of the steepest declines in premature mortality observed in recent history across countries and a dramatic increase in the uptake of maternal and child health interventions.^1^

A core component of Vision 2020 is the establishment of a large, diverse, and competent health workforce. However, by 2011 Rwanda’s ratio of health professionals to general population of 0.72/1000 was still falling significantly short of the World Health Organization’s (WHO’s) recommended (at the time) target of 2.3/1000.^2^ The majority of practicing physicians, nurses, and midwives were lacking any kind of formal postgraduate degree while a shortage of faculty and limited infrastructure, equipment, and supplies in the country’s health graduate schools and teaching hospitals were preventing Rwanda from increasing the output and improving the quality of the existing training programs. It is to address the health workforce shortage and strengthen the capacity of its health graduate schools that the Government of Rwanda decided to launch the Human Resources for Health Program in Rwanda (HRH Program) in 2012.

### The Rwanda Human Resources for Health Program

The HRH Program is an innovative and ambitious 7-year health professional training initiative led by the Government of Rwanda and funded by the US President’s Emergency Plan for AIDS Relief through the Centers for Disease Control and Prevention (CDC) and the Global Fund to Fight AIDS, Tuberculosis, and Malaria (Global Fund) with an initial budget of approximately US$150 million.^3^ The key features of the HRH Program are the product of broader changes and progress in the fields of global health and health workforce education over the past ten years.^4-11^ Many of these features are aligned with the United Nations’ Sustainable Development Goals, are shared with other US Government-funded health professional training initiatives in sub-Saharan Africa, and include: (*a*) alignment with responsiveness to local priorities; (*b*) country ownership; (*c*) strong stakeholder coordination; (*d*) funding flexibility; (*e*) competency-based training and pedagogic innovation; (*f*) institutional capacity strengthening; and (*g*) long-term sustainability.^4,12-16^ Similar features are also shared with the WHO’s Global Strategy for Human Resources for Health,^17^ the WHO’s Guidelines for Transformative Education for Health Professionals,^18^ and the 5-Year Action Plan for Health Employment and Inclusive Economic Growth by the Organization for Economic Co-Operation and Development, the International Labor Organization, and the WHO.^19^

**Figure 1:**
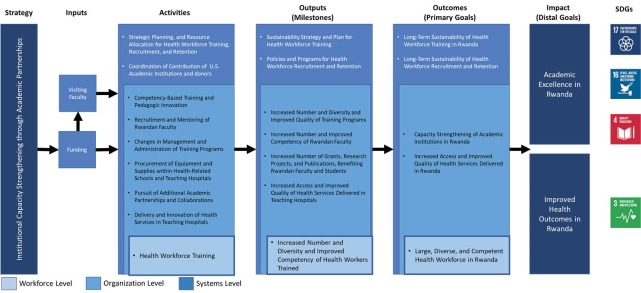
Theoretical Framework for the Evaluation of the Human Resources for Health Program.*
* Adapted from PEPFAR’s theoretical framework for capacity building and strengthening.

The primary goal of the HRH Program is to train a large, diverse, and competent health workforce in Rwanda. The HRH Program also seeks to strengthen the capacity of academic institutions in Rwanda to sustain the training programs initiated and supported by the HRH Program by: (*a*) increasing the number and competencies of Rwandan faculty; (*b*) strengthening the capacity of non-academic domains (such as management and administration or equipment and supplies) within the Ministry of Health (MoH), health-related schools, and teaching hospitals; and (*c*) pursuing additional academic partnerships and collaborations between Rwandan academic institutions and US academic institutions (Figure 1). This article is an organizational case study of the HRH Program which aims to: (*a*) present the activities conducted and milestones achieved by the program; (*b*) describe the challenges encountered during the program’s implementation; and (*c*) share good practices adopted and lessons learned by those implementing the program since 2012.

### A New Strategy for Capacity Strengthening and for Academic Partnerships

**Figure 2:**
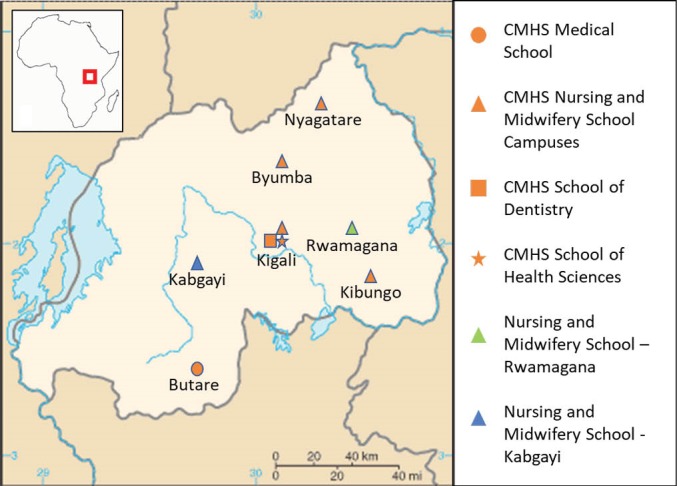
Location of Health-Related Schools in Rwanda Participating in the Human Resources for Health Program.

Since 2012, a consortium of US academic institutions has been deploying visiting faculty to the College of Medicine and Health Sciences (CMHS) at the University of Rwanda and to the Nursing and Midwifery Schools in Rwamagana and Kabgayi. The CMHS was established in 2013 by bringing together different health-related schools under a single institutional umbrella and is comprised of 5 schools: the School of Medicine and Pharmacy (which is based in Butare, Rwanda’s second largest city), the School of Nursing and Midwifery (which has 4 separate campuses across the country), the School of Dentistry, the School of Public Health, and the School of Health Sciences (all of which are based in Kigali, Rwanda’s capital) (Figure 2).

The US academic institutions were selected by the MoH either because they had already been working effectively for years in Rwanda or because they had already expressed an interest in working in Rwanda and had established reputations as leaders in global health for their respective disciplines or clinical specialties. Because the HRH Program follows a tied-aid model, no academic institutions from countries other than the United States have joined the academic consortium since 2012.

The visiting faculty are deployed to Rwanda for 2 to 12 months each year depending on departmental needs and specialty areas. Shorter deployments of 2 to 3 months generally correspond with sub-specialty areas while longer deployments of 6 to 12 months correspond with most clinical specialties and health-related disciplines. Upon graduation, the best performing Rwandan students are expected to be recruited as faculty by the CMHS and gradually replace the visiting faculty.

**Figure 3:**
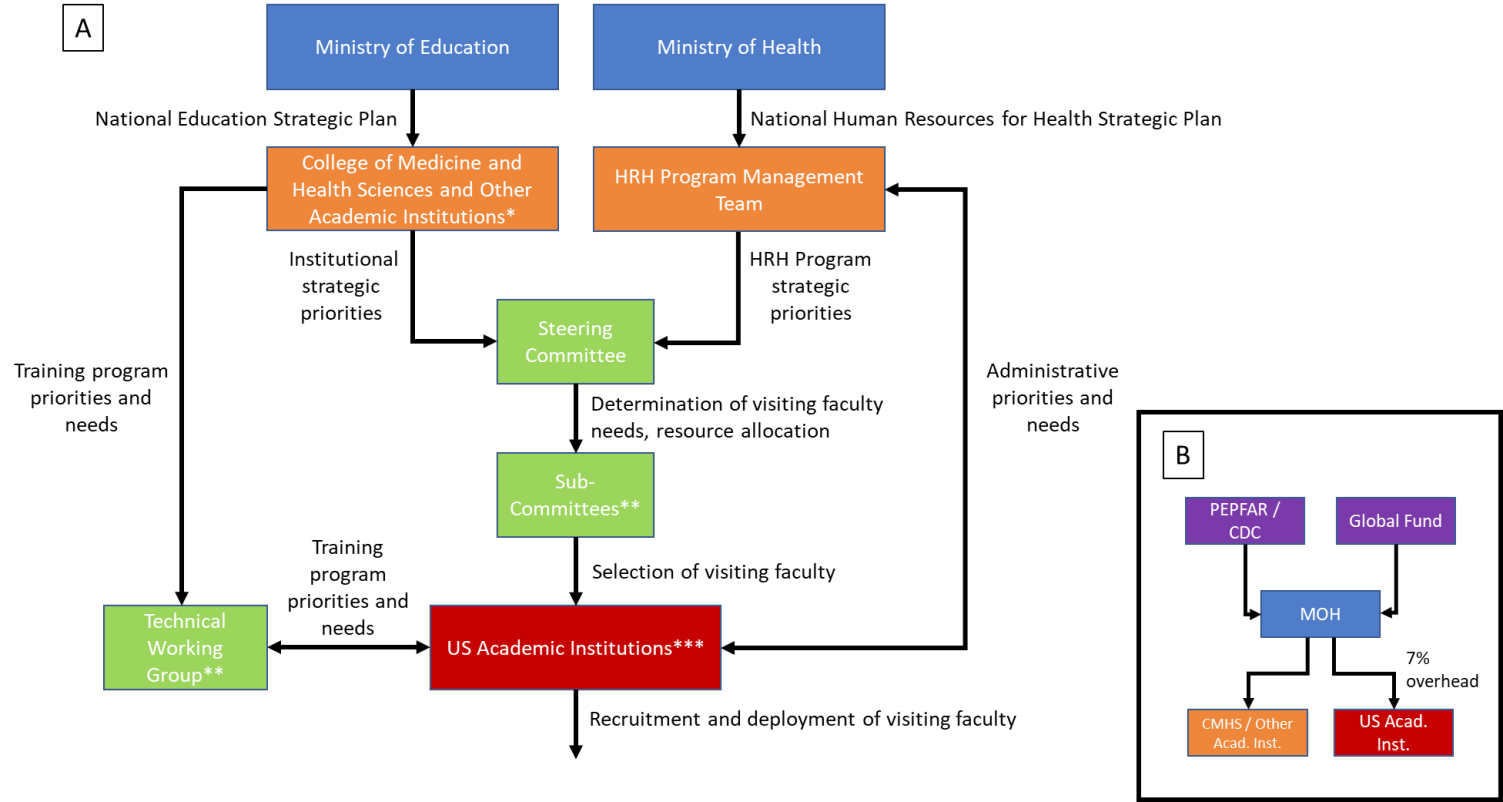
Governance Structure and Decision-Making Process (A); and Funding Flow (B) for the Human Resources for Health Program.
* College of Medicine and Health Sciences (CMHS) and Other Institutions: School of Dentistry, School of Health Sciences, School of Medicine and Pharmacy, School of Nursing and Midwifery, School of Public Health. The Schools of Nursing and Midwifery in Rwamagana and Kabgayi participated in the Human Resources for Health Programs Program as well but remained independent institutions from the CMHS while still being part of the University of Rwanda.

The contributions of the US academic institutions are coordinated by the HRH Program management team within the MoH to maximize synergy and avoid gaps and overlaps in the resources and expertise provided to the CMHS (Figure 3A). A steering committee comprised of leaders from the MoH and from the CMHS (which reports to the Ministry of Education) convenes at the end of each academic year to determine the number of visiting faculty needed for the following academic year. Four health professional sub-committees chaired by Deans of the Schools within the CMHS (one for medicine, one for nursing and midwifery, one for oral health, and one for health management and implementation) then convene to review the profiles of the candidates identified by the US academic institutions for the visiting faculty positions and select the stronger candidates. After the US academic institution receive notification of the candidates selected by the sub-committees, the visiting faculty are recruited and deployed to Rwanda.

** Sub-Committees and Working Groups: Medicine, Nursing and Midwifery, Oral Health, Health Management and Implementation.

*** US Academic Institutions: *Medicine* **-** Albert Einstein College of Medicine at Yeshiva University, Beth Israel Deaconess Hospital, Boston Children’s Hospital, Brigham and Women’s Hospital, Brown University (University Emergency Medicine Foundation, University Medicine Foundation, Rhode Island Hospital), Columbia University Mailman School of Public Health, Duke University School of Medicine (2012-2015), Geisel School of Medicine at Dartmouth, Massachusetts Eye and Ear Infirmary, Massachusetts General Hospital, University of Maryland School of Medicine, University of Texas Medical Branch (no faculty deployed, 2012-2016), University of Virginia School of Medicine, Yale School of Medicine; *Nursing and Midwifery**-*** Duke University School of Nursing (2012-2015), Howard University Division of Nursing, New York University College of Nursing, University of Illinois at Chicago College of Nursing, University of Maryland School of Nursing (2012-2017), University of Texas Health Sciences Center at Houston (no faculty deployed, 2012-2016); *Oral Health** - ***Harvard School of Dental Medicine, University of Maryland School of Dentistry; *Health Management and Implementation** - ***Yale University Global Health Leadership Institute, Harvard Medical School Department of Global Health and Social Medicine.

With support from The ELMA Foundation (an international philanthropic organization) the Clinton Health Access Initiative assisted the MoH in the first 2 years of the HRH Program with program design and the development of administrative, financial, and human resources policies and procedures. Since 2014, the MoH has had complete ownership of the HRH Program and is responsible for coordination and communication among stakeholders.

Unlike other US bilateral and multilateral global health grants in low-income countries, funds for the HRH Program flow from the donors directly to the MoH rather than through an intermediary or directly to the US academic institutions (Figure 3B).^3^ The HRH Program was designed so that the MoH would utilize approximately 60% of the funds to cover salary, benefits, travel, and housing for visiting faculty through reimbursements to US academic institutions. The remaining funds are used to improve the equipment and supplies within the CMHS and the teaching hospitals and to support the HRH Program management team within the MoH. All of the US academic institutions agreed to charge an overhead rate of only 7% of their direct costs instead of the much higher rates usually charged for research grants to ensure that the majority of funds available are devoted to cover implementation costs in-country rather than to cover US-based administrative costs. While both the CDC and Global Fund began funding the HRH Program in 2012, the CDC (which covered approximately 70% of the costs) decided to cease funding after March 2017 because of a change in strategy which prioritized a more direct alignment between funding allocation and the pursuit of eradication of HIV/AIDS in Rwanda.

## Methods

The authors decided to adopt an organizational case study design, which they believed would not be hindered by the limited amount of data collected in the years preceding this study and would be compatible with a post-hoc evaluation of the HRH Program. In conducting this study, the authors followed the guidelines and the checklist from the National Institute for Health Research for organizational case studies available at: https://www.journalslibrary.nihr.ac.uk/hsdr/hsdr04010#/table11.

### Data Sources

The data for this organizational case study were collected through a variety of sources and methodologies including official reports from the MoH and the US academic institutions, databases from the HRH Program management team within the MoH, databases from the registrar’s office of the CMHS, and minutes from meetings and conference calls. Additionally, the authors were organized into working groups according to their discipline and clinical specialty and asked to complete a 63-item survey to capture descriptive data related primarily to the activities conducted and non-numerical milestones achieved by the HRH Program within the following programmatic domains: (*a*) Competency-Based Training and Pedagogic Innovations; (*b*) Changes in Governance and Administration of Training Programs; (*c*) Procurement of Equipment and Supplies; (*d*) Recruitment, Retention, and Career Development of Health Workforce in Rwanda; (*e*) Recruitment and Career Development of Rwandan Faculty; (*f*) Establishment of Additional Academic Partnerships and Collaborations. No ethics review approval was deemed to be necessary by the leaderships of the MoH and of the CMHS, based on their institutional requirements and policies, as the data collection and analysis for this manuscript fell under the category of “program evaluation.”

### Data Analysis

The current number of graduates for most of the training programs initiated or supported by the HRH Program was estimated by analyzing the student graduation databases for each health-related discipline until 2017. The projected number of graduates by 2019 was inferred by adding the projected number of graduates for 2018 and 2019 to the estimated current number of graduates until 2017. The projected number of graduates for 2018 and 2019 was inferred by multiplying number of students enrolled for the annual attrition rate estimated for each one of the training programs (5% across all the master’s degrees for physicians, 5% for the advanced diplomas in nursing and midwifery, 2% for the bachelor’s degrees in nursing and midwifery, 5% for the oral health training programs, and 10% for the health management and implementation training programs). For the Nursing and Midwifery Schools in Rwamagana and Kabgayi no student enrollment data was available at the time of the analysis. Therefore, the average number of graduates per year between 2013 and 2016 for the training programs offered by these institutions was used to infer the projected number of graduates for 2017, 2018, 2019. No correlation analysis was performed because of the difficulty of accounting for all additional variables linked to an individual milestone. Additionally, no analysis was performed to directly link the implementation of the HRH Program to more distal metrics of success such as improved health outcomes both within the teaching hospitals and across the health system.

## Results: Activities Conducted and Milestones Achieved by the Human Resources for Health Program Since 2012

### Increased Number and Diversity of Health Workforce Trained

**Figure 4:**
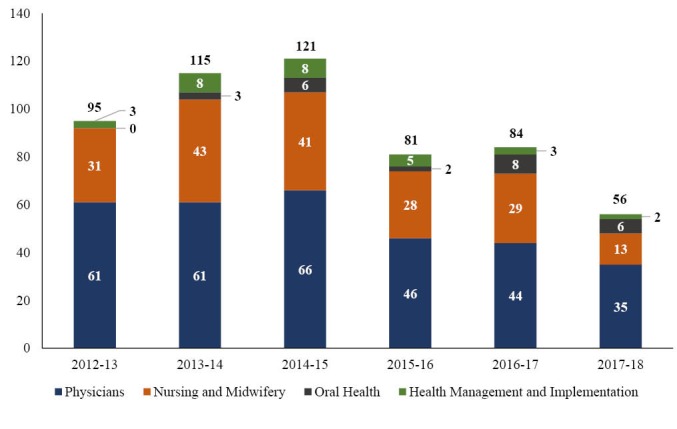
Visiting Faculty Deployment across 4 Health-Related Disciplines (2012-2017)*.
* Data for years 2012-2013, 2013-2014, and 2014-2015 presented in the Mid-Term Review of the Rwanda Human Resources for Health Program released by the Ministry of Health in 2016.

Since the launch of the HRH Program in 2012, 22 training programs (12 of which did not exist previously) have been supported across 4 health-related disciplines (medicine, nursing and midwifery, oral health, and health management and implementation) (Table 1). In the first 5 years, US academic institutions have deployed an average of 99 visiting faculty per year across these disciplines and different clinical specialties and sub-specialties (Figure 4).

**Table 1:** Estimated Cumulative Current and Projected Cumulative Number of Graduates Across 22 Training Programs during the Human Resources for Health Program (2013-2019)

**Training Program**	**Year Launched**	**Program Duration**	**Cumulative Current Graduates 2013-2017**	**Cumulative Projected Graduates 2013-2019**
**Physicians**				
Master of Medicine in General Surgery	2006	4	15	31
Master of Medicine in Internal Medicine	2006	4	53	85
Master of Medicine in Obstetrics and Gynecology	2006	4	35	56
Master of Medicine in Pediatrics	2006	4	32	57
Master of Medicine in Anesthesiology	2007	4	13	24
Master of Medicine in Otorhinolaryngology	2010	4	8	12
Master of Medicine in Emergency Medicine	2013	4	0	12
Master of Medicine in Neurosurgery	2013	6	1	4
Master of Medicine in Orthopedic Surgery	2013	6	0	5
Master of Medicine in Pathology	2013	4	4	9
Master of Medicine in Psychiatry	2013	4	3	7
Master of Medicine in Urology	2013	6	2	3
Master of Medicine in Radiology	2016	4	0	0
**Nursing and Midwifery**				
Advanced Diploma in Nursing (A1)	2007	3	1677	2199
Advanced Diploma in Midwifery (A1)	2007	3	803	1092
Bachelor of Science in Nursing (A0)	2007	4	206	293
Bachelor of Science in Midwifery (A0)	2013	2	91	108
Master of Science in Nursing^a^				
*Critical care and trauma nursing*	2010	2	20	35
*Nursing education leadership and management*	2015	2	13	26
*Medical surgical*	2015	2	16	33
*Neonatology*	2015	2	12	25
*Nephrology*	2015	2	7	13
*Oncology*	2015	2	7	15
*Pediatrics*	2015	2	19	37
*Perioperative*	2015	2	13	25
**Oral Health**				
Bachelor of Science in Dental Therapy	2009	4	181	231
Bachelor of Science in Dental Surgery	2012	5	0	31
**Health Management and Implementation**				
Master of Hospital and Healthcare Administration	2013	2	51	66
Master of Global Health Delivery^b^	2015	2	24	63
**Total**			3306	4598

^a^116 students enrolled since 2015, first class graduated in 2017. ^b^Moved from the School of Public Health within the College of Medicine and Health Sciences to the new University of Global Health Equity.

The majority of visiting faculty recruited and deployed to Rwanda have been graduates of North American academic institutions. In some instances (primarily for nursing and midwifery) the US academic institutions have also recruited and deployed visiting faculty from other sub-Saharan African countries, in order to ensure that the proper mix of technical knowledge and skills and understanding of local context and priorities would be available to stakeholders in Rwanda. Based on the analysis of 64 visiting faculty deployed by a sub-set of 7 academic institutions participating in the HRH Program (Harvard Medical School, Harvard School of Dental Medicine, Brigham and Women’s Hospital, Boston Children’s Hospital, Massachusetts General Hospital, Massachusetts Eye and Ear Infirmary, and Beth Israel Deaconess Medical Center), approximately 56% of visiting faculty were mid-career at the time of deployment (6-19 years since completion of training), 38% were junior (<5 years) and 22% senior (>20 years). Approximately 55% of the visiting faculty were deployed to Rwanda for six months or longer, and 52% returned to Rwanda at least one more year after their first year of deployment.

**Figure 5:**
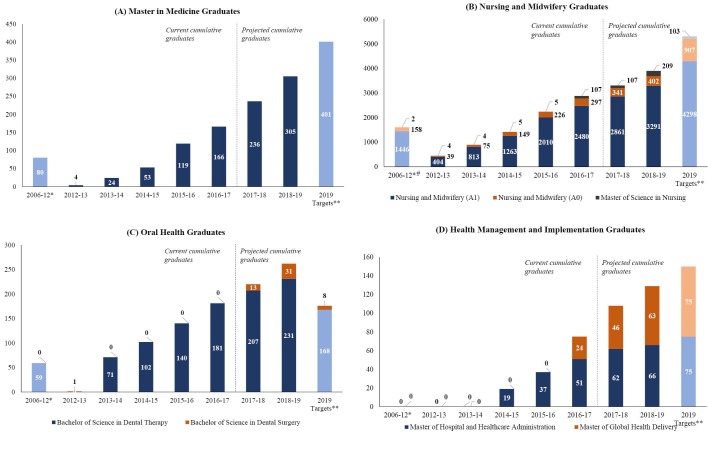
Current and Projected Cumulative Number of Graduates across 4 Health-Related Disciplines (A, B, C, D) During the Human Resources for Health Program (2013-2019).
* Before Human Resources for Health Program.

The Schools at the CMHS have been able to increase the number of students enrolled across the 22 training programs initiated or supported by the HRH Program because of the increased number of faculty available through the US academic institutions. As a result, the HRH Program is on track to graduate an estimated total of 4598 students across these programs (Table 1); a significant increase from the number of students who graduated from these programs in the 7 years preceding the HRH Program (Figure 5). While the number of students projected to graduate by 2019 appears to be close to and in the case of oral health surpass the original targets, it is important to clarify that the original targets of the HRH Program represented the number of new health professionals joining the health workforce and not the number of new graduates (which is what is reported in this study) (Figure 5). Differences between the projected number of graduates and the original targets can be explained in part by the de-prioritization of certain training programs and the new prioritization of others.

** Original targets of Human Resources for Health Program.

^#^Data from Nursing and Midwifery Schools in Rwamagana and Kabgayi is missing.

For the discipline of medicine, the training programs which were deprioritized (master’s degrees in family medicine and community health, neurology, and oncology) led to a total loss of 84 new physicians from the original target of 401 new physicians with specialty training, while the training programs which were newly prioritized (master’s degrees in neurosurgery and urology) added only 7 new physicians to the same original target (Figure 5A). The MoH decided to de-prioritize master’s degree in family medicine and community health for 2 main reasons: (*a*) because of the need to devote the limited resources and expertise to the newly prioritized training programs; and (*b*) because new medical graduates opting to join the health workforce as general practitioners (rather than pursue specialty training) were felt to already possess the proper knowledge and skills required to deliver basic medical, surgical, and obstetrical services.

The de-prioritization of the A1 (advanced diploma) to A0 (bachelor’s degree) nursing bridging program led to a loss of approximately 500 new nurses from the original target of 907 new nurses and midwives with bachelor’s degree. The MoH decided to de-prioritize the A1 to A0 nursing bridging program to devote additional resources and expertise to the 8 different tracks within the newly established master’s degree in nursing (critical care and trauma, nursing education, leadership and management, medical surgical, neonatology, nephrology, oncology, pediatrics, perioperative). In the case of the A2 (entry level) to A1 (advanced diploma) nursing and midwifery bridging program it was difficult for the School at the CMHS to sustain the high enrollment rates necessary to fully meet the ambitious original targets although the increase in training output remained substantial (Figure 5B). Student enrollment in newly established private schools rather than the School at the CMHS might also explain part of the difference between projected number of graduates and original targets for the discipline of nursing and midwifery.

For oral health, the higher number of projected graduates is explained by an effort by the School at the CMHS to meet new targets selected by the MoH for dental therapists and dental surgeons, which were higher than those originally selected for the HRH Program (Figure 5C). For health management and implementation, the lower number of projected graduates is in part explained by the new prioritization of a master’s degree in global health delivery (which is on track to graduate 63 students by 2019) over an originally planned certificate degree in health administration (which was expected to graduate at least 75 students over the 7 years of the HRH Program) (Figure 5D).

### Development of Policies and Programs for Health Workforce Career Development and Retention

To ensure their retention within the health workforce, and in exchange for government sponsorship during their training years, the MoH has enforced a mandatory 4- to 5-year contract of employment in the public sector for all new graduates. Additionally, visiting faculty have also supported the development and implementation of continuing professional development programs for practicing health professionals across the 4 health-related disciplines (Box 1).

**Box 1. Development of Health Policies and Programs for Health Workforce Career Development, and Retention**

- Mandatory 4- or 5-year contracts for new graduates to work in public sector
- Continuing professional development programs and sessions for practicing health professionals
- Physicians: cardiopulmonary resuscitation, child abuse, critical care, external and middle ear diseases, gastroenterology, hematology, emergency medicine and acute care, infectious diseases, malnutrition, maxillofacial trauma, neonatology, non-communicable diseases, oral health, palliative care, regional anesthesia
- Nurses and Midwives: advanced life support, basic life support, critical care, diabetes, evidence-based decision making, infection control, myocardial infarction, oral health, patient assessment and chart documentation, palliative care, viral hepatitis
- Oral health professionals: evidence-based decision making, infection control, oral surgery, periodontology, prosthodontics
- Health administrators and implementers: executive leadership, global health delivery

### Institutional Capacity Strengthening

#### Recruitment and Career Development of Rwandan Faculty

Between January 2012 and March 2017, 24 new Rwandan faculty and 31 tutorial assistants have been recruited by the Schools at the CMHS, 22 faculty have been recruited by the Schools of Nursing and Midwifery in Rwamagana and Kabgayi. As of March 2017, approximately 80 additional Rwandan faculty were expected to be recruited by the CMHS no later than by the end of the 2019-2020 academic year (Table 2). The rate of recruitment might decrease if the MoH decides to extend the duration of the HRH Program for 3 to 4 years beyond 2019 to more gradually replace visiting faculty with Rwandan faculty. In the teaching hospitals, approximately 60 new Master of Medicine graduates have been recruited and issued contracts requiring them to devote 20% of their time to training. Over the next 2 academic years, additional Master of Medicine graduates will be issued these contracts and the MoH is now considering adopting a similar approach for nursing and midwifery graduates as well.

**Table 2:** Institutional Capacity Strengthening

**Recruitment and Professional Development Rwandan Faculty**
• 24 new Rwandan faculty and 31 tutorial assistants recruited by the CMHS while additional 22 faculty recruited by the Nursing and Midwifery Schools in Rwamagana and Kabgayi between 2012 and 2016 • 60 Master of Medicine graduates employed by teaching hospitals in Kigali and Butare issued contracts with 20% of time devoted to mandatory teaching responsibilities • CMHS to recruit approximately 80 additional Rwandan faculty no later than by the end of the 2019- 2020 academic year according to the college’s sustainability strategy framework document • Continuing professional development programs in research, grant writing, and mentorship and supervision across the majority training programs
**Pursuit of Additional Academic Partnership and Collaborations**
• Small grants and donations secured for training, research, and health service delivery activities, faculty and student exchange programs, and international travel including: • 11 Master of Medicine students participated in clinical clerkships in the United States • 7 Master of Medicine students and 2 oral health students presented their work at international and regional conferences • At least 20 faculty gave lectures in the United States or presented their work at international and regional conferences • Additional funding secured to expand and equip pathology laboratory and build telepathology capacity at Rwanda Military Hospital in Kigali • New income generated for the School of Dentistry by the renovated oral health clinic • Research center established at the teaching hospital in Kigali • Access to UpToDate® and other online training materials in teaching hospitals in Kigali and Butare • Research and evaluation projects • *Physicians*: factors influencing choice of anesthesia as a career by physicians; epidemiology of injuries and trauma; revision of acute respiratory distress syndrome case definition; assessment of intensive care unit mortality; assessment of maternal near miss and mortality in tertiary care; management of post-Caesarean section peritonitis; assessment of perioperative mortality rate; adherence to treatment for pediatric HIV infection; assessment of radiology capacity in Rwanda; assessment of surgery curriculum for physicians; prevalence and clinical presentation of breast cancer; prevalence of antibiotic resistance in neonatology; challenges of HIV care in adolescents; assessment of simulation training for cardiopulmonary resuscitation • *Nurses and midwives*: assessment of E-learning nursing education in Rwanda; assessment of interventions to improve hand washing and medical records completion by nurses and midwives; labor monitoring by midwives; and rates of intravenous catheter infections in neonatology • *Oral health professionals*: national oral health survey and development and assessment of research ethics curriculum • *Health administrators and implementers:* establishment and assessment of human resources tracking system; risk factors for homicide victimization; assessment of twinning program in the HRH Program; support to research and evaluation projects of physicians, nurses and midwives, and oral health professionals • Over 80 scientific articles published by Rwandan faculty and students with support from faculty from US academic institutions

Abbreviations: CMHS, College of Medicine and Health Sciences; HRH Program, Human Resources for Health Program.

Recruitment of Rwandan faculty has required inter-sectoral partnership. The Schools at the CMHS, in the University of Rwanda, which reports to the Ministry of Education, have developed policies and procedures and secured funds to recruit new Rwandan faculty. Simultaneously, the MoH, which manages the HRH Program and developed the national strategy for health workforce development, has allowed the Schools at the CMHS to recruit some of these graduates as faculty rather than deploying them throughout the country.

The HRH Program has strengthened the knowledge and skills of both Rwandan and visiting faculty by establishing a twinning program (the formal pairing of faculty around shared academic responsibilities and interests). Since 2012, Rwandan faculty have collaborated with visiting faculty on a variety of training, research, and health service delivery activities and have also benefited from continuing professional development programs developed and implemented with the support of the visiting faculty (Table 2).

#### Pursuit of Additional Academic Partnerships and Collaborations

There are 19 US academic institutions participating in the HRH Program as of April 2017 (ranging from a minimum of 16 to a maximum of 24). Of the 5 academic institutions that have opted to leave the HRH Program since 2012, 2 did so because of changes in their departmental priorities, one because of the heavy administrative burden of managing their contribution to the program, and 2 because of ongoing challenges with the invoicing and reimbursement processes related to the program. The training, research, and health service delivery activities conducted by Rwandan and visiting faculty have led to more than 80 scientific publications (Table 2).^20-100^ Rwandan faculty and students have traveled to the United States to give lectures, participate in clinical clerkships, and pursue further training, while others have presented their work at national, regional, and international conferences. These activities have been funded by additional grants, not by the HRH Program, and highlight the importance of the additional academic partnerships and collaborations established since 2012. The US academic institutions have also benefited significantly from their participation in the HRH Program by providing opportunities to their own faculty and students to engage and pursue careers in global health and to learn and apply innovative approaches to pedagogy and health service delivery.^16^

#### Increased Number and Diversity and Improved Quality of Training Programs

As mentioned above, the HRH Program in 2012 has supported a total of 22 training programs (9 of which already existed and 12 of which did not exist previously) across the health-related disciplines of medicine, nursing and midwifery, oral health, and health management and implementation. While increasing training output, the HRH Program has also sought to improve the quality of the training programs through: (*a*) competency-based training and pedagogic innovation; (*b*) stronger management and administration of the training programs; and (*c*) improvements in equipment and supplies within the Schools at the CMHS and the teaching hospitals (Table 3).

**Table 3:** Increased Number and Diversity and Improved Quality of Training Programs

**Competency-Based Training and Pedagogic Innovation**
• Enhanced mentorship and supervision of students across all training programs during: • Inpatient and outpatient care • Quality improvement projects • Research projects, publications, and dissertations • New pedagogic approaches across all training programs, including: • Simulation-based training • Flipped classroom and case-based approach • Revision of existing curricula and development of new modules and clinical rotations • Research methodology curriculum for Master of Medicine students, nursing and midwifery students, and oral health students • Bridging programs for: • Physicians (from post-graduate diploma to Master of Medicine in Internal Medicine and in Emergency Medicine) • Nursing and midwifery students from A2 (entry) to A1 (advanced diploma) level • Midwifery students from A1 (advanced diploma) to A0 (bachelor’s degree) level • Dental therapy students from A1 (advanced diploma) to A0 (bachelor’s degree) level • Online curricula, modules, and training materials • Inter-professional training for: • Master of Medicine students (Internal Medicine, Pediatrics, Radiology, and Surgery) • Medical students, nursing and midwifery students, and oral health students • Health administration and global health delivery students
**Changes in Management and Administration of Training Programs**
• Associate heads of departments established for Master of Medicine programs across all clinical specialties • Position of Director of Master of Science in Nursing established • New administrators recruited by Schools and Departments at the CMHS • Monthly case reports via teleconference between Rwanda and United States for master’s degree in anesthesiology • New School of Dentistry established at CMHS • Grand rounds, morning reports, morbidity and mortality reports, and journal clubs established across multiple training programs • Classroom and practicum rotation schedules revised and enforced across all training programs • Core competencies and evaluation practices for students revised and standardized across multiple training programs • Chief residency program established for Master of Medicine (Internal Medicine, Obstetrics and Gynecology, Pediatrics) • Task-shifting of teaching responsibilities to senior Master of Medicine students across multiple training programs • Increased graduation rates documented for Master of Medicine students (medicine, pediatrics), and dental therapy students • >85% of surveyed Master of Medicine students satisfied with the acquisition of clinical competencies across all clinical specialties and credited the HRH Program for the improvement in their learning experience
**Procurement of Equipment and Supplies within Health-Related Schools and Teaching Hospitals**
• Simulation laboratories equipped at University hospitals in Kigali and Butare and at School of Nursing and Midwifery in Kigali • Preclinical skills laboratory and oral health clinic renovated at School of Dentistry • Computers, modems, and teleconferencing equipment procured at CMHS and at teaching hospitals in Kigali and Butare • Medical equipment (anesthesia machines, infusion pumps, laparoscopy equipment, monitors, incubators, ultrasound machines, ventilators, etc) procured at teaching hospitals in Kigali and Butare

Abbreviations: CMHS, College of Medicine and Health Sciences; HRH Program, Human Resources for Health Program.

## Increased Access and Improved Quality of Health Services Delivered in Teaching Hospitals

The HRH Program is designed to link the improvement in the quality of training to the quality of health service delivery in teaching hospitals in a way that mutually reinforces both, for example, by emphasizing enhanced mentoring and supervision at point of care over classroom teaching. The HRH Program has also invested in equipment and supplies within the teaching hospitals in Kigali and Butare to strengthen their capacity to provide practicing health professionals with the proper tools to deliver health services efficiently and effectively. Lastly, Rwandan faculty and visiting faculty have worked together to develop and implement new clinical protocols and programs; assess gaps; and evaluate governance, administration, and other policies related to specific clinical programs and diseases (Table 4).^22,26,34-36,38,47,50,81,82,91-100^

**Table 4:** Increased Access and Improved Quality of Health Services Delivered in Teaching Hospitals

**New Clinical Protocols and Programs**
• Re-organization of the emergency medicine department at teaching hospital in Kigali with regionalized care, introduction of triage based on symptom severity, and establishment of intensive care unit • Decrease of mortality in emergency medicine department from 6.8% to 1.2% • Clinical protocols in anesthesia, airway reconstruction, asthma and pneumonia management, diabetic ketoacidosis in children, electrolyte imbalances in children, emergency medicine, epilepsy management, intensive care, post-partum infectious complications, sepsis management • Inpatient consultation services in cardiology, nephrology, and palliative care • Establishment of surgical specialty and sub-specialty teams (acute care surgery, elective general surgery, neurosurgery, orthopedics, pediatric general surgery, plastics surgery, urology) with regionalized post-operative care units, and dedicated intra-operative care teams • Expansion of the intensive care units at the public teaching hospitals and establishment of high-dependency units (“step-down units”) • Establishment of tumor boards to discuss cancer treatment plans with multi-disciplinary teams • Outreach by the multi-disciplinary surgical teams from teaching hospitals to provincial and district hospital to address the backlog of surgical cases and provide mentoring and supervision at point of care in surgery, anesthesia, and nursing • Establishment of telepathology services at teaching hospital in Kigali • Strengthening of microbiology laboratory services with the development of hospital-based antibiograms to guide choice of antibiotics • Community outreach efforts by nursing and midwifery faculty and students in primary care and non-communicable diseases • Community outreach efforts by oral health faculty and students
**Governance, Administration, and Policies**
• Revision of medical records and establishment of patient databases across clinical specialties • Triage protocols to improve patient flow in emergency medicine, primary care, and operating room • Decrease of ratio between emergency surgical cases and elective surgical cases from 4/1 to 1/2 and decrease in wait time for elective surgery cases from 12 months to 30 days at teaching hospital in Kigali • Decrease in average pathology reporting time from 6 weeks to 10 days at teaching hospital in Kigali • Safe surgery checklist in teaching hospitals • Infection control and antibiotic stewardship protocols and campaigns in teaching hospitals • National policies for emergency medicine, non-communicable diseases, palliative care, and pathology

## Discussion

### Strengths and Weaknesses of the Study

The diverse and non-systematic nature of data sources and methodologies to collect data likely resulted in under-reporting of activities and milestones. Student enrollment and graduation databases across schools within and outside (Schools of Nursing and Midwifery in Rwamagana and Kabgayi) the CMHS were often inconsistent and incomplete and extensive work went into reconciling differences and finding missing information (especially to determine the number of nurses and midwives who graduated since 2013). In retrospect, the resources and expertise available for monitoring and evaluation of the HRH Program were limited and did not enable the MoH and the other stakeholders to perform the needed systematic and in-depth evaluation of the HRH Program’s impact compared to the baseline. The prioritization in the budget (both by the MoH and the donors) of implementation costs, the simultaneous under-estimation of the costs associated with other critically important programmatic components (including not only management and administration, but also monitoring and evaluation), and the unprecedented scale and complexity of the HRH Program are the main reasons for the mismatch between needs and availability of resources and expertise. In the future, should health professional training initiatives similar to the HRH Program be implemented in other low-income countries, greater attention should be devoted to the establishment of a strong monitoring and evaluation platform. Additionally, all large-scale multi-year global health programs or interventions should include an implementation research component. Post-hoc evaluations conducted a few years after the launch of these programs or interventions, such as the one conducted for this study, are not designed to provide ongoing data-driven feedback to implementers, which is necessary to learn new lessons and improve the quality of implementation in real-time.

Linking scale-up and improvement in the quality of training to improvements in the quality of health service delivery and in health outcomes (both within the teaching hospitals and across the health system) has been especially challenging. Additionally, the 63-item survey utilized to collect descriptive data did not include open-ended questions on perceived successes, opportunities, and challenges related to the HRH Program. As a result, the authors did not perform a thematic analysis (or other types of qualitative research analysis) to identify recurrent themes in the responses provided by the co-authors or other participants in the HRH Program and consolidate them in an empirically validated list of lessons learned. The authors believe that the HRH Program would greatly benefit from a properly resourced external evaluation, which would look beyond the program’s activities and milestones presented in this article and focus instead on the program’s outcomes and more distal metrics of success (especially as they relate to improved health outcomes). The same evaluation could also integrate a quantitative component with a comprehensive and detailed qualitative component through a mixed-methodology study design.

### Challenges Encountered, Good Practices Adopted, and Lessons Learned

Despite the study limitations, important good practices have been adopted and lessons have been learned by the authors (Table 5) to guide the implementation of similar health professional training initiatives in other low-income countries in the future. It is important to note that these practices and lessons are the product of the authors’ own reflections as implementers and not the result of a thematic analysis of the data captured by the 63-item survey described in the methods section or of other qualitative research methodologies. Overall, the achievements of the HRH Program have been substantial, although some challenges persist.

**Table 5:** Lessons Learned During the First 5 Years of the Rwanda Human Resources for Health Program

**Recruitment and Deployment of Visiting Faculty**
• Recruitment and deployment of visiting faculty might require flexibility. Under the right circumstances, shorter durations of deployment for senior faculty and recruitment of junior faculty are feasible. • Twinning between local and visiting faculty should be centered around shared academic interests and its success evaluated based on the achievement of specific objectives.
**Funding**
• While recognizing the importance of keeping funds devoted to coordination and communication low, there must be sufficient funds to cover the real cost of managing complex and multilayered initiatives such as the HRH Program. • Restrictions on how funds may be spent, year-by-year renewal, or earlier than planned withdrawal of donor commitment can negatively impact implementation. Greater funding flexibility and multi-year commitments are required for successful implementation.
**Sustainability**
• A very deliberate strategy should be created to ensure that the resources and expertise of foreign academic institutions are leveraged to strengthen the capacity of local academic institutions. Sustainability of impact needs to be actively pursued from the outset of initiatives such as the HRH Program. • Retention of local graduates as faculty and investments in their career development are critical to sustain the training programs supported by initiatives such as the HRH Program. • Strengthening the capacity of local academic institutions across non-academic domains (such as management and administration, finance, fundraising and development, etc) is a critical component of a sustainability strategy. • Diversification of funding sources from single large donors to multiple smaller donors and decentralization of fund-raising to smaller groups of participants and stakeholders are critical to achieve sustainability.

Abbreviation: HRH, Human Resources for Health.

Recruitment of visiting faculty was initially hindered by a limited pool of full-time senior and mid-career candidates, especially for the discipline of medicine, who were preferred by the Schools at the CMHS and by the students. However, if carefully selected and adequately mentored, junior visiting faculty can be equally effective while shorter durations of deployment for senior visiting faculty can be considered if continuity over multiple years is possible. Additionally, some visiting faculty initially struggled to transition to clinical practice in resource-limited settings and adapt to different professional habits and cultural norms. Serial revisions in the orientation training and the presence of a core group of returning visiting faculty have improved this transition but more needs to be done. For example, the HRH Program would benefit from the availability of more comprehensive and updated orientation materials that visiting faculty could review online prior to their deployment to Rwanda.

Twinning between Rwandan faculty and visiting faculty has worked quite well at times. However, mismatched seniority levels, technical expertise, and academic interests between faculty twins, and difficulties protecting time for Rwandan faculty, as well as formal processes to evaluate and improve twinning, have been persistent challenges.^88^ Thus, leadership at the MoH and the CMHS recommended a revision of the twinning program in 2016 in which Rwandan and visiting faculty identify and conduct activities of shared academic interest and meet the objectives of the Schools at the CMHS and are then formally evaluated based on their ability to achieve these objectives. However, to this day, the revised twinning program has not been yet implemented broadly or uniformly across all Schools and Departments within the CMHS.

Ensuring coordination and communication among many stakeholders has been difficult, especially in the early years of the HRH Program. It has taken several years to find the right balance between the role of the MoH, which manages the HRH Program and is the primary interlocutor with the US academic institutions, and the Schools at the CMHS, which are the primary recipients of the visiting faculty but whose line Ministry is the Ministry of Education. More importantly, both the Schools at the CMHS and the US academic institutions received insufficient funding for management and administration, which would have allowed them to improve communication and coordination and provide better mentorship and supervision to the visiting faculty. Additional funding would have enabled US based coordinators to travel to Rwanda to support the visiting faculty and establish closer ties with the academic leadership at the CMHS. Since 2012, coordination and communication has generally improved as meetings and conference calls with working groups for health disciplines and clinical specialties (with representatives from the MoH, the Schools at the CMHS, and US academic institutions) are scheduled more frequently, although such improvement has been inconsistent, especially in the last 12 months, and challenges persist.

Despite the positive impact of improvements in equipment and supplies within the Schools at the CMHS and the teaching hospitals, these have been insufficient to entirely address the existing gaps. Additionally, the Schools at the CMHS and the teaching hospitals have lacked the resources and expertise necessary to install and maintain the newly procured equipment which has led to delays and inconsistencies in its utilization. The MoH is now working to strengthen its supply-chain management, installation, and upkeep capacity.

The flow of funds from donors to the MoH rather than directly to the US academic institutions has facilitated country ownership and alignment of the HRH Program to local priorities. Despite this novel funding model,^3,4^ management of the funds has at times been challenging. Mismatches between fiscal years for the CDC, the Global Fund, the CMHS, and US academic institutions has made it difficult to process payments in a timely manner and track expenditures. Furthermore, funds have come with spending restrictions on critical items such as monitoring and evaluation, equipment, and supplies while renewal of donor commitment has occurred on a year-by-year basis and late within the previous academic year. These delays have hindered recruitment efforts from US academic institutions as numerous ideal candidates eventually have opted to seek other employment opportunities rather than wait until funds were secured.

Lastly, the decision by the CDC to cease funding for the HRH Program after March 2017 put a strain on the MoH, the CMHS, and its US partners and has led to approximately a 30% reduction in the number of full time equivalents for visiting faculty deployed to Rwanda for the 2017-2018 academic year compared to what had been originally planned. The number of visiting faculty to be deployed in the 2018-2019 academic year is yet to be finalized but will likely be similar. In the future, greater flexibility in funding expenditures and longer-term commitment from donors will be needed to ensure that initiatives such as the HRH Program achieve their full potential.

### The Path to Sustainability

As this organizational case study demonstrates, the resources and expertise available through the HRH Program have already strengthened the capacity of the Schools at the CMHS.

However, the sustainability of these achievements will need to be pursued by all the stakeholders even more deliberately and aggressively. As a result, the academic leadership of the CMHS has finalized a sustainability strategy framework document with goals and objectives to strengthen the capacity of the Schools to sustain the training programs initiated or supported by the HRH Program. More detailed planning will now be needed to determine the precise amount of new Rwandan faculty to be recruited (and outline the process to recruit them), secure additional funds, and establish additional partnerships and collaborations with the US academic institutions that will continue after the HRH Program formally comes to an end.

To ensure programmatic sustainability, the HRH Program was designed to gradually replace the visiting faculty with newly recruited Rwandan faculty, to be selected among the new graduates of the training programs initiated or supported by the program. While the transition between visiting faculty and Rwandan faculty has already begun, this effort will need to be scaled up substantially before the end of the HRH Program. Funding available through the Global Fund was utilized to maintain a core group of 56 visiting faculty in Rwanda for the 2017-2018 academic year and will be utilized to maintain a similar number of visiting faculty for at least one more academic year. Simultaneously, some of the responsibility for teaching junior students will also be delegated to adequately mentored and supervised senior students. Additionally, the MoH is considering extending funding for the HRH Program for 3 to 4 years beyond 2019 to ensure a more gradual transition between visiting faculty and Rwandan faculty.

Once recruited, the new Rwandan faculty will need to be adequately supported by the Schools at the CMHS and by the visiting faculty, especially in the early stages of their career. The establishment of additional academic partnerships and collaborations with the US academic institutions and eventually the launch of additional training programs (for example in sub-specialty areas) will be critical to ensure that the newly recruited Rwandan faculty are able to further develop professionally and thrive academically.

The Schools will also have to further strengthen their capacity across several non-academic domains, such as management and administration, finance, human resources, fund-raising and development. These domains are essential to a high-performing institution but are often overlooked by health professional training initiatives. In retrospect, even though the HRH Program sought to strengthen these non-academic domains, not enough resources and expertise where devoted to them. More importantly, when the HRH Program was first designed, not enough planning went into comprehensively and systematically determining the extent to which these domains needed to be strengthened and the timeline by which this would happen.

Regarding the financial sustainability of the HRH Program, the cost of recruiting and deploying visiting faculty and procuring equipment and supplies within the Rwandan academic institutions accounted for the largest proportion of the overall budget. These costs were the highest in the earlier years of the HRH Program, when higher numbers of visiting faculty were needed in-country and the gaps in equipment and supplies were the greatest. The same costs have since decreased significantly now that Rwandan faculty have begun to replace visiting faculty and most of the planned improvements in equipment and supplies have been achieved.

The primary costs that the MoH will need to sustain beyond the duration of the HRH Program will be those of the salaries for the increased numbers of Rwandan physicians, nurses, midwives, oral health professionals, and health managers that have joined or are expected to join the public sector. When the HRH Program was first designed, the determination was that the MoH would not need any external funding to cover these costs because of the projected increase in Rwanda’s gross domestic product (GDP) and in the percentage of government spending devoted to the health sector. This determination has not changed.

The costs that the Rwandan academic institutions will need to sustain beyond the duration of the HRH Program will be primarily those of the salaries of the newly recruited Rwandan faculty but also the costs of subsidizing tuition fees for increasing numbers of students, further improving equipment and supplies, and further strengthening management and administration and other non-academic domains. While the Ministry of Education and the Schools at the CMHS have devoted additional funds to cover some of these costs, more funding will be necessary to achieve financial sustainability. Diversifying funding sources beyond the CDC and the Global Fund, strengthening the capacity of the CMHS to generate soft money through grants, and decentralizing fund-raising to individual schools and departments at the CMHS and their US partners will be critical. The schools at the CMHS and some of their US partners are already seeking additional funding opportunities, submitting proposals, and in some cases securing funds to sustain their work. However, these efforts will need to be scaled up substantially before the end of the HRH Program.

## Conclusion

The Rwanda HRH Program is an innovative and ambitious initiative that has already substantially scaled up and improved the quality of health professional training in Rwanda.^3^ Despite some persistent challenges, important lessons have been learned and good practices adopted by the MoH of Rwanda, the Schools at the CMHS, and a consortium of 19 US academic institutions participating in the HRH Program. The authors believe that the HRH Program can serve as a model for other initiatives that seek to address the shortage of qualified health professionals in low-income countries and strengthen the long-term capacity of local academic institutions. Liberia and Sierra Leone, where weak health systems were devastated by the Ebola epidemic,^101^ as well as other sub-Saharan African countries have expressed an interest in adopting a similar model. To realize their full potential and achieve sustainable impact, initiatives like the HRH Program require strong communication and coordination, adequate policies and procedures to recruit visiting faculty and support them once deployed, significant investment in the recruitment and career development of local faculty, adequate resources devoted to management and administration or infrastructure and equipment, and greater funding flexibility and long-term commitment from donors.

## Acknowledgements

The authors would like to acknowledge the hundreds of visiting faculty who participated in the HRH Program and the leaders and administrators of the MoH, the Schools at the CMHS, and the US academic institutions who rendered the implementation of the HRH Program possible. The authors would also like to acknowledge Laetitia Nyirazinyoye and Libby Abbott who conducted the majority of data collection and analysis for the Mid-Term Review of the HRH Program (mentioned in Figure 4). Dr. Corrado Cancedda conceptualized the manuscript, wrote the first draft, led the development of the manuscript, and successfully completed the submission of the manuscript when he was affiliated to the Division of Global Health Equity at Brigham and Women’s Hospital (Boston, MA, USA) and the Department of Global Health and Social Medicine at Harvard Medical School (Boston, MA, USA).

## Ethical issues

No ethics review approval was deemed to be necessary by the leaderships of the MoH and of the CMHS (based on their institutional requirements) and by representatives of the US academic institutions in the core working group for this article as the data collection and analysis for this manuscript fell under the category of “program evaluation.”

## Competing interests

Authors declare that they have no competing interests.

## Funding

This work was supported by The United States President’s Emergency Plan for AIDS Relief through the Centers for Disease Control and Prevention funded the HRH Program from July 2012 until March 2017; The Global Fund to Fight AIDS, Tuberculosis and Malaria, has been funding the HRH Program since July 2012; The ELMA Foundation provided funding for early planning and management and administration assistance of the HRH Program between 2011 and 2013 and for the preparation of this article between January 2015 and December 2016; and The Abundance Foundation provided funding for the preparation of the article between April 2016 and the date of submission.

## Authors’ contributions

CC and AgB conceptualized the manuscript, wrote the first draft, and led the development of the manuscript. JS, LVA, PC, RR, PEF, RoR, and SR provided feedback on first draft and supported initial development of the manuscript. EsN, JS, and LM provided data and feedback from the Rwanda Ministry of Health. CM, DKT, DoM, JK, PK, and SR provided data and feedback from the leadership of the College of Health Sciences, University of Rwanda. The following authors provided data and feedback on the manuscript related to their respective area of contribution to the Human Resources for Health Program: ACL, CDM, JEO, MD, RTM, and TT for Anesthesiology and Emergency Medicine; AG, CM, JB, and VM for Oral Health; BH-G, DKT, EK, JR, and ReW for Health Management and Implementation; AR, CC, JD, LVA, NT, PEP, ReR, TDW, and VD for Internal Medicine; BB, CAB, DC, DoM, LLM, MadM, MarM, MH, MR, RTK, TLH for Nursing and Midwifery; LB-M, RoW, SR, UM for Obstetrics and Gynecology; BR, DaM for Pathology; JCK, KW, LA, MC, MiK, NM, TR for Pediatrics; GR and SK for Psychiatry; LK and SS for Radiology; AlB, AmB, EmN, FN, JFC, MuK, RG, and RoR for Surgery; MM for 7 Harvard-affiliated institutions; and AS, IM, MeK, PO, and TN for support during HRH program design and initial implementation. AY and JM edited and provided feedback on the manuscript; collected, processed, analyzed data; and supported the development of the manuscript.

## Authors’ affiliations

^1^Center for Global Health, Perelman School of Medicine, University of Pennsylvania, Philadelphia, PA, USA. ^2^Office of the Vice-Chancellor, University of Rwanda, Kigali, Rwanda. ^3^Rwanda Human Resources for Health Program Team, Ministry of Health, Kigali, Rwanda. ^4^Office of the Dean, School of Medicine and Pharmacy, College of Medicine and Health Sciences, University of Rwanda, Kigali, Rwanda. ^5^Center for Surgery and Public Health, Brigham and Women’s Hospital, Boston, MA, USA. ^6^Department of Global Health and Social Medicine, Harvard Medical School, Boston, MA, USA. ^7^Center for Health Equity, Geisel School of Medicine, Dartmouth College, Hanover, NH, USA. ^8^Department of Medicine, Geisel School of Medicine, Dartmouth College, Hanover, NH, USA. ^9^Division of Global Health Equity, Department of Medicine, Brigham and Women’s Hospital, Boston, MA, USA. ^10^Office of the Principal, College of Medicine and Health Sciences, University of Rwanda, Kigali, Rwanda. ^11^Department of Surgery, Faculty of Clinical Medicine and Dentistry, Kampala International University - Western Campus, Ishaka, Uganda. ^12^School of Nursing and Midwifery, College of Medicine and Health Sciences, University of Rwanda, Kigali, Rwanda. ^13^Office of the Dean and Department of Oral and Maxillofacial Surgery, Oral Pathology and Oral Medicine, School of Dentistry, College of Medicine and Health Sciences, University of Rwanda, Kigali, Rwanda. ^14^School of Health Sciences, College of Medicine and Health Sciences, University of Rwanda, Kigali, Rwanda. ^15^Department of Anesthesiology, School of Medicine and Pharmacy, College of Medicine and Health Sciences, University of Rwanda, Kigali, Rwanda. ^16^Department of Ear, Nose, and Throat, School of Medicine and Pharmacy, College of Medicine and Health Sciences, University of Rwanda, Kigali, Rwanda. ^17^Department of Internal Medicine, School of Medicine and Pharmacy, College of Medicine and Health Sciences, University of Rwanda, Kigali, Rwanda. ^18^School of Medicine and Public Health, Faculty of Health and Medicine, University of Newcastle, Newcastle, NSW, Australia. ^19^Department of General Medicine, Calvary Mater Hospital, Newcastle, NSW, Australia. ^20^Department of Neurosurgery, School of Medicine and Pharmacy, College of Medicine and Health Sciences, University of Rwanda, Kigali, Rwanda. ^21^Department of Obstetrics, Gynecology, and Reproductive Sciences, Yale School of Medicine, New Haven, CT, USA. ^22^Department of Orthopedic Surgery, Rwanda Military Hospital, Kigali, Rwanda. ^23^Department of Pathology, School of Medicine and Pharmacy, College of Medicine and Health Sciences, University of Rwanda, Kigali, Rwanda. ^24^Department of Pediatrics, School of Medicine and Pharmacy, College of Medicine and Health Sciences, University of Rwanda, Kigali, Rwanda. ^25^Department of Mental Health, School of Medicine and Pharmacy, College of Medicine and Health Sciences, University of Rwanda, Kigali, Rwanda. ^26^Department of Radiology, School of Medicine and Pharmacy, College of Medicine and Health Sciences, University of Rwanda, Kigali, Rwanda. ^27^Department of Surgery, School of Medicine and Pharmacy, College of Medicine and Health Sciences, University of Rwanda, Kigali, Rwanda. ^28^Yale School of Medicine, New Haven, CT, USA. ^29^Department of Pediatrics, Icahn School of Medicine at Mount Sinai, New York City, NY, USA. ^30^Department of Pediatrics, BronxCare Health System, Bronx, NY, USA. ^31^Department of Pediatrics, Yale School of Medicine, New Haven, CT, USA. ^32^Division of Hospital Medicine, Department of Pediatrics, Children’s Hospital Los Angeles, Los Angeles, CA, USA. ^33^Department of Pediatrics, Keck School of Medicine, University of Southern California, Los Angeles, CA, USA. ^34^Global Health Leadership Institute, Yale School of Public Health, New Haven, CT, USA. ^35^Department of Preventive and Community Dentistry, School of Dentistry, College of Medicine and Health Sciences, University of Rwanda, Kigali, Rwanda. ^36^University of Global Health Equity, Kigali, Rwanda. ^37^Clinton Health Access Initiative, Boston, MA, USA. ^38^Clinton Health Access Initiative, Kigali, Rwanda. ^39^Office of Global and Community Health, Harvard School of Dental Medicine, Boston, MA, USA. ^40^Department of Oral Health Policy and Epidemiology, Harvard School of Dental Medicine, Boston, MA, USA. ^41^Department of General Pediatrics, Boston Children’s Hospital, Boston, MA, USA. ^42^Department of Emergency Medicine, Warren Alpert Medical School of Brown University, Providence, RI, USA. ^43^Department of Medicine, Warren Alpert Medical School of Brown University, Providence, RI, USA. ^44^Department of Pediatrics, Warren Alpert Medical School of Brown University, RI, USA. ^45^sidHARTe Program, Heilbrunn Department of Population and Family Health, Mailman School of Public Health, Columbia University, New York City, NY, USA. ^46^Department of Emergency Medicine, Columbia University College of Physicians and Surgeons, New York City, NY, USA. ^47^Department of Anesthesiology, Geisel School of Medicine, Dartmouth College, Hanover, NH, USA. ^48^Dartmouth-Hitchcock Medical Center, Lebanon, NH, USA. ^49^Department of Pediatrics, Geisel School of Medicine, Dartmouth College, Hanover, NH, USA. ^50^Department of Surgery, Geisel School of Medicine, Dartmouth College, Hanover, NH, USA. ^51^Duke Hubert-Yeargan Center for Global Health, Durham, NC, USA. ^52^Department of Medicine, Duke University School of Medicine, Durham, NC, USA. ^53^Duke Global Health Institute, Durham, NC, USA. ^54^Duke University Medical Center, Durham, NC, USA. ^55^Duke University School of Nursing, Durham, NC, USA. ^56^Department of Obstetrics & Gynecology and Women’s Health, Albert Einstein College of Medicine, New York City, NY, USA. ^57^Obstetrics & Gynecology and Women’s Health, Montefiore Medical Center, New York City, NY, USA. ^58^Division of Nursing, Howard University College of Nursing and Allied Health Sciences, Washington, DC, USA. ^59^University of Connecticut School of Nursing, Storrs, CT, USA. ^60^New York University Rory Meyers College of Nursing, New York City, NY, USA. ^61^University of Illinois at Chicago College of Nursing, Chicago, IL, USA. ^62^Columbia University School of Nursing, New York City, NY, USA.^63^Department of Family & Community Health, University of Maryland School of Nursing, Baltimore, MD, USA. ^64^Global Education and Mentorship, Office of Global Health, University of Maryland School of Nursing, Baltimore, MD, USA. ^65^Department of Oncology & Diagnostic Sciences, University of Maryland School of Dentistry, Baltimore, MD, USA. ^66^Office of Global Health, University of Maryland School of Nursing, Baltimore, MD, USA. ^67^Partnerships, Professional Education, and Practice, University of Maryland School of Nursing, Baltimore, MD, USA. ^68^Department of Anesthesiology, University of Virginia School of Medicine, Charlottesville, VA, USA. ^69^Department of Anesthesiology Perioperative and Pain Medicine, Boston Children’s Hospital, Boston, MA, USA. ^70^Department of Surgery, University of Virginia School of Medicine, Charlottesville, VA, USA. ^71^Center for Global Health, American Society for Clinical Pathology, Chicago, IL, USA. ^72^Department of Immunology and Infectious Diseases, Harvard T. H. Chan School of Public Health, Boston, MA, USA. ^73^Department of Psychiatry, Boston Children’s Hospital, Boston, MA, USA. ^74^Department of Radiology, Brigham and Women’s Hospital, Boston, MA, USA. ^75^Global Health and Research Programs, Biomedical Research Institute, Brigham and Women’s Hospital, Boston MA, USA. ^76^Department of Internal Medicine, Yale School of Medicine, New Haven, CT, USA. ^77^Institute for Health Policy and Clinical Practice, Dartmouth College, Hanover, NH, USA. ^78^Office of the Vice-Chancellor, University of Global Health Equity, Kigali, Rwanda.

## Key messages

**Implications for policy makers**

- Multi-year training initiatives based on partnerships between academic institutions in high- and low-income countries can help address the global shortage of health professionals.
- Expatriate local faculty will initially do most of the teaching but can gradually be replaced by new graduates from the training programs supported by such initiatives.
- Funding flexibility (without excessive restriction on how funds can be spent) and reliable funding availability over multiple years are critical to ensuring the success of such initiatives.
- Sustainability can be achieved through institutional capacity strengthening, which encompasses investments in both academic (ie, teaching, research) as well as non-academic (ie, management and administration, finance, fundraising and development, etc) domains.
- The establishment of additional partnerships and collaborations between academic institutions in high- and low-income countries is also critical for sustainability.

**Implications for the public**

Health professional training initiatives like the Human Resources for Health Program (HRH Program) in Rwanda, funded by the US Government and the Global Fund for a total budget of approximately US$150 million over 7-years, can help address the global shortage of health professionals. In the first 5 years since the launch of the HRH Program in 2012, a consortium of US academic institutions has deployed an average of 99 faculty per year to support and/or launch 22 training programs, which are on track to train almost 4600 health professionals (physicians, nurses, midwives, oral health professionals, health administrators) by 2019. To ensure sustainability, new graduates from the training programs supported by the HRH Program have begun to gradually replace US faculty. The HRH Program has also served as a catalyst for additional academic partnerships and collaborations between US and Rwanda academic institutions. These partnerships will also play a critical role in achieving sustainability by promoting faculty career development and by diversifying funding for academic activities in Rwanda.
